# Will There Ever Be Cure for Chronic, Life-Changing Colistin-Resistant *Klebsiella pneumoniae* in Urinary Tract Infection?

**DOI:** 10.3389/fmed.2021.806849

**Published:** 2021-12-24

**Authors:** Aye Mya Sithu Shein, Parichart Hongsing, Shuichi Abe, Sirirat Luk-in, Naveen Kumar Devanga Ragupathi, Dhammika Leshan Wannigama, Tanittha Chatsuwan

**Affiliations:** ^1^Department of Microbiology, Faculty of Medicine, Chulalongkorn University, King Chulalongkorn Memorial Hospital, Thai Red Cross Society, Bangkok, Thailand; ^2^Antimicrobial Resistance and Stewardship Research Unit, Faculty of Medicine, Chulalongkorn University, Bangkok, Thailand; ^3^Interdisciplinary Program of Medical Microbiology, Graduate School, Chulalongkorn University, Bangkok, Thailand; ^4^Mae Fah Luang University Hospital, Chiang Rai, Thailand; ^5^School of Integrative Medicine, Mae Fah Luang University, Chiang Rai, Thailand; ^6^Department of Infectious Diseases and Infection Control, Yamagata Prefectural Central Hospital, Yamagata, Japan; ^7^Department of Clinical Microbiology and Applied Technology, Faculty of Medical Technology, Mahidol University, Bangkok, Thailand; ^8^Biofilms and Antimicrobial Resistance Consortium of ODA Receiving Countries, The University of Sheffield, Sheffield, United Kingdom; ^9^Department of Chemical and Biological Engineering, The University of Sheffield, Sheffield, United Kingdom; ^10^Department of Clinical Microbiology, Christian Medical College, Vellore, India; ^11^School of Medicine, Faculty of Health and Medical Sciences, The University of Western Australia, Nedlands, WA, Australia

**Keywords:** *Klebsiella pneumoniae*, urinary tract infection, colistin-resistant, colistin-resistant *Klebsiella pneumoniae*, chronic infection, biofilm infections, chronic urinary infection

## Introduction

Urinary tract infection (UTI) is a considerable public health issue that threatens 150 million individuals globally each year, exhibiting a significant impact on the economy and quality of life in affected individuals (1, 2). UTI is especially prevalent among females, both in terms of occurrence and recurrence (2). Bacterial colonization and subsequent invasion in various parts of the urinary tract, combined with biofilm formation, induce uncomplicated mild UTI, chronic recurrent UTI, and complicated severe UTI which can lead to septicemia and renal failure, resulting in mortality rates of 20–40% among patients with underlying immunocompromised conditions, long-term urinary catheterization, and chronic kidney diseases (2–6).

*Klebsiella pneumoniae* is one of the most common causal microorganisms that causes UTI in clinical settings (7). Their abilities to construct biofilms in medical devices, including urinary catheters, as their critical step in their disease pathogenesis can result in biofilm-mediated antibiotic tolerance in these pathogens (7, 8). Moreover, worldwide emerging trends of multidrug-resistant (MDR) and pandrug-resistant (PDR) strains in *K. pneumoniae* make it challenging for clinicians to provide prompt and efficient therapy, imposing a considerable negative burden on patient's morbidity and mortality (9, 10). The growing incidences of antimicrobial resistance and biofilm-mediated antimicrobial tolerance in *K. pneumoniae* with limited treatment alternatives, combined with the failure to discover new antibiotics have triggered the reappraisal of colistin as a valid therapeutic option (11).

Colistin is a bactericidal cationic antibiotic that triggers increased membrane permeabilities and cell death via electrostatic interactions with lipid A of lipopolysaccharide (LPS) (11, 12). However, colistin-resistant *K. pneumoniae* has been continuously raised from <2 to 9% worldwide as a result of the current surge in colistin treatment administration (13, 14). Infections with colistin-resistant *K. pneumoniae* were also revealed to be independently associated with the excess of mortality in healthcare settings (15).

**Figure 1:**
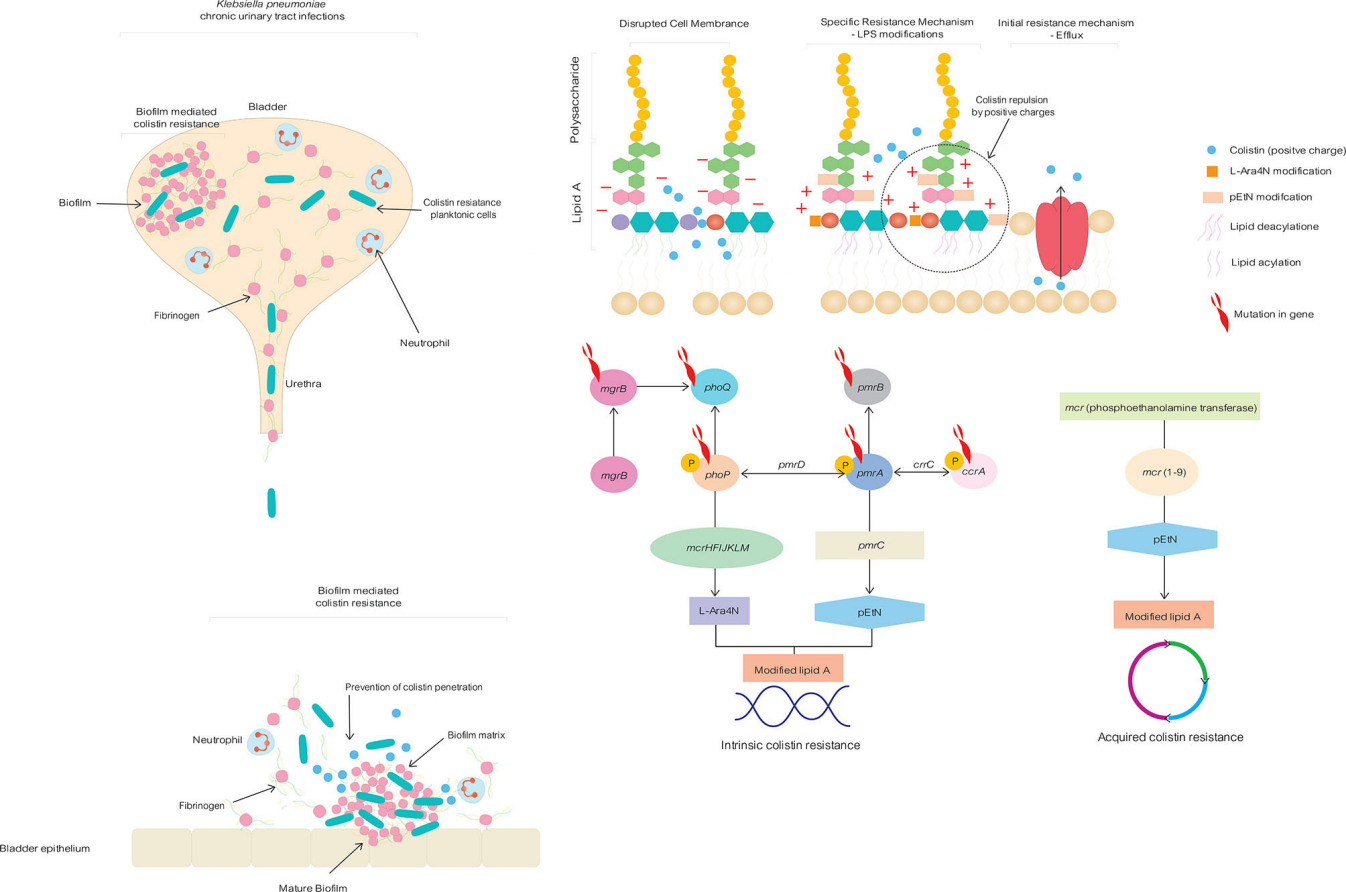
Intrinsic chromosome-mediated and acquired plasmid-mediated colistin resistance mechanisms in *Klebsiella pneumoniae togeetrh* with biofilm formation in chronic, life-changing urinary tract infection.

The intrinsic chromosome-mediated and acquired plasmid-mediated alteration of lipid A phosphate moieties in LPS with amino-4-deoxy-L-arabinose (L-Ara4N) and phosphoethanolamine (pEtN) weakens the electrostatic affinity of colistin to LPS, causing colistin resistance in *K. pneumoniae* (Figure 1) (10). The therapeutic efficacy of colistin become compromised as a consequence of increased genetic mutation and the dissemination of plasmid-mediated *mcr* genes, emphasizing the importance of exploring innovative alternative treatment strategies to address untreatable colistin-resistant *K. pneumoniae* related with UTI (Table 1). There for in this opinion we discuss potential effective treatment options for patients who suffer from chronic colistin-resistant *Klebsiella pneumoniae* urinary tract infection.

**Table 1:** Different therapeutic strategies to confront colistin resistance in chronic, life-changing colistin-resistant *Klebsiella pneumoniae* urinary tract infection.

**Drugs**	**Single therapy/combination therapy**	**Mechanism of action**	**Antibacterial spectrum**	**Dosage**	**Pharmacokinetic properties**	**Advantage and efficacy**	**Trial stage**	**References**
**Novel antibiotic therapies**								
Plazomicin (Aminoglycoside)	Single therapy	Bactericidal drug that inhibit bacterial protein synthesis	Multidrug-resistant Gram-negative ESBL-producing, carbapenem-resistant and colistin-resistant *Enterobacteriaceae* with *mcr1* and *pmrAB* or *phoPQ* or *mgrB* mutations	Intravenous 30-min infusion of single dose plazomicin (15 mg/kg) but modification of dosage to 10 mg/kg is needed in patients with lower creatinine clearance.	• Plazomicin is entirely eliminated by renal tubular excretion. • Compared to patients with normal renal function, reduced Volume of distribution at steady state (Vss) and Total clearance (CLT) were observed in patients with impaired renal function.	Excellent treatment response rates with favorable safety and tolerability profiles	Phase III	(16, 17)
Meropenem-vaborbactam (Carbapenem-β lactamase inhibitor)	Single therapy	Bactericidal drug that targets to bind directly catalytic serine residues to inhibit β-lactamases activities	Multidrug-resistant Gram-negative ESBL-producing, carbapenem-resistant *Enterobacteriaceae*	Intravenous 3-h infusion of 4 g (2 g meropenem/2 g vaborbactam) every 8 h, but modification of dose is needed in patients with lower creatinine clearance.	• Similar linear pharmacokinetic profiles of both meropenem and vaborbactam • Both drugs are entirely eliminated by renal tubular excretion. • Compared to patients with normal renal function, reduced Volume of distribution at steady state (Vss) and Total clearance (CLT) were observed in patients with impaired renal function.	Excellent treatment response rates with favorable safety and tolerability profiles	Phase III	(18–20)
Cefiderocol (Siderophore cephalosporin)	Single therapy	Bactericidal drug with enhanced outer membrane penetration ability using iron transport system and inhibit bacterial cell wall synthesis	Gram-negative ESBL-producing, carbapenem-resistant *Enterobacteriaceae*	Intravenous infusion of single or multiple doses (2 g) every 8 h, but modification of dose is needed in patients with lower creatinine clearance.	• Cefiderocol is entirely eliminated by renal tubular excretion. • Compared to patients with normal renal function, reduced Volume of distribution at steady state (Vss) and Total clearance (CLT) were observed in patients with impaired renal function.	Excellent treatment response rates with favorable safety and tolerability profiles	Phase III	(21, 22)
**Combination antibiotic therapies**								
Colistin	+ Aminoglycosides (Amikacin, Gentamicin, and Tobramycin) + Carbapenems (Imipenem and Meropenem) + Cephalosporins (Cefoperazone, Cefotaxime, Ceftazidime and Ceftolozane and Tazobactam) + Fluoroquinolones (Ciprofloxacin, Moxifloxacin) + Fosfomycin + Tetracyclines (Minocycline) + Penicillin (Piperacillin)	Bactericidal	Colistin-resistant *K. pneumoniae*	Not applicable	Not applicable	Not applicable	Pre-clinical (*in vitro*)	(23)
Colistin	+ Sulfadiazine	Bactericidal	Colistin-resistant *K. pneumoniae*	Not applicable	Not applicable	Not applicable	Pre-clinical (*in vitro*)	(24)
Colistin	+ Minocycline + Linezolid + Fusidic acid + Levofloxacin	Bactericidal	Colistin-resistant *Enterobacteriaceae*	Not applicable	Not applicable	Not applicable	Pre-clinical (*in vitro*)	(25)
Colistin	+ Ertapenem + Meropenem	Bactericidal	Colistin-resistant *K. pneumoniae*	Not applicable	Not applicable	Not applicable	Pre-clinical (*in vitro*)	(26)
Ceftazidime-avibactam	+ Avibactam	Bactericidal	Colistin-resistant and carbapenemase-producing *K. pneumoniae*	Not applicable	Not applicable	Not applicable	Pre-clinical (*in vitro*)	(27)
Gentamicin	+ Tigecycline	Bactericidal	Carbapenem-resistant and colistin-resistant *K. pneumoniae*	Combined gentamicin and tigecycline therapy during the first 5 days after diagnosis and maintained for at least 5 days.	Not applicable	Improve treatment success rates and clinical outcomes of patients	Retrospective cohort study	(28)
Colistin	+ Fosfomycin	Bactericidal	Extensively and pandrug-resistant *K. pneumoniae*	Intravenous Fosfomycin with 24 g/day median dose for a median of 14 days, in combination with colistin	Not applicable	Better bacterial eradication with improved clinical outcomes in critically ill patients	Multicenter prospective study	(29)
Gentamicin	+ Fosfomycin and Tigecycline	Bactericidal	Colistin-resistant *K. pneumoniae*	Intravenous single daily dose of Gentamicin 5 mg/kg combined with intravenous Tigecycline at loading dose of 100–200 mg followed by 50–100 mg every 12 h and Fosfomycin 4 g every 6 h for 10–14 days	Not applicable	Improve survival of patients	Prospective cohort study	(30)
Ertapenem	+ Doripenem or Meropenem	Bactericidal	Pandrug-resistant *K. pneumoniae*	Ertapenem 1 g every 24 h + Doripenem 2 g every 8 h or Meropenem 1 g every 8 h for a total of 10–20 days	Not applicable	Effective therapeutic response with no recurrence	Case study	(31)
**Non-antibiotic drugs in combination therapies**								
Azidothymidine (Antiretroviral drug)	+ Colistin	Bactericidal	Gram-negative colistin-resistant *Enterobacteriaceae* including colistin-resistant *K. pneumoniae* and *Escherichia coli* with *mcr1*	Intravenous infusion of colistin 4,2 and 2 MIU together with 200, 100, and 100 mg of Azidothymidine	Low dose regimen (Azidothymidine with Colistin 2 MIU) with no observed renal toxicities.	Favorable therapeutic combination for overcoming colistin resistance in *Enterobacteriaceae*	Phase I	(32–35)
EDTA (Metal ions chelator)	+ Colistin	Bactericidal	Colistin-resistant *K. pneumoniae*	EDTA 12 mg/ml with Colistin 1 μg/ml	Not applicable	Not applicable	Pre-clinical (*in vitro*)	(36)
Pentamidine (Antiprotozoal drug)	+ Aminoglycosides + Tigecycline + Doripenem, + Rifampicin	Bactericidal	Colistin-resistant *Enterobacteriaceae*	Not applicable	Not applicable	Not applicable	Pre-clinical (*in vitro*)	(37, 38)
Mitotane (Chemotherapeutic drug)	+ Polymyxin B	Bactericidal	Polymyxin-resistant *K. pneumoniae*	Mitotane (4 mg/L) with Polymyxin B (2 mg/L)	Not applicable	Not applicable	Pre-clinical (*in vitro*)	(39)
Closantel (Antihelminth drug)	+ Polymyxin	Bactericidal	Polymyxin-resistant *A. baumannii*	Closantel (4–16 mg/L) with polymyxin B (2 mg/L)	Not applicable	Not applicable	Pre-clinical (*in vitro*)	(40)
Niclosamide (Parasiticidal drug)	+ Colistin	Bactericidal	Colistin-resistant *K. pneumoniae* with *mgrB* & *pmrB* mutation	Niclosamide (2 μM) with Colistin (sub-MIC)	Not applicable	Not applicable	Pre-clinical (*in vitro*)	(41)
Tamoxifen, Raloxifen, and Toremifen (Selective estrogen receptor modulators)	+ Colistin	Bactericidal	Polymyxin-resistant *K. pneumoniae*	Tamoxifen (8 mg/L) with Polymyxin B (0.5 mg/L)	Not applicable	Not applicable	Pre-clinical (*in vitro*)	(42)
**Natural compounds in combination therapies**								
Pterostilbene (Potent *mcr1* inhibitor)	+ Polymyxin	Bactericidal	*mcr* variants-positive *Enterobacteriaceae*	Pterostilbene (80 mg/kg) with Polymyxin B (5 mg/kg)	Not applicable	Not applicable	Pre-clinical (*in vitro*)	(43)
Resveratrol (Natural chemical of many plants)	+ Colistin	Bactericidal	Colistin-resistant *K. pneumoniae* with *mgrB* & *pmrB* mutation	Resveratrol (128 mg/L) with Colistin (2 mg/L)	Not applicable	Not applicable	Pre-clinical (*in vitro*)	(44)
Eugeno l (Natural chemical of plants that bind Mcr1)	+ Colistin	Bactericidal	Colistin-resistant *E. coli* with *mcr1*	Not applicable	Not applicable	Not applicable	Pre-clinical (*in vitro*)	(45)
**Additional therapies**								
Bacteriophage therapy	Single therapy	Bactericidal	Colistin-resistant *K. pneumoniae* with *mcr1* and *mgrB* mutations	Not applicable	Not applicable	Not applicable	Pre-clinical (*in vitro*)	(46)
Nanoparticle encapsulation of colistin for targeted delievery	Single therapy	Bactericidal	Drug-resistant *K. pneumoniae*	Not applicable	Not applicable	Not applicable	Pre-clinical (*in vitro*)	(47)

## Novel Antibiotic Therapies to Overcome Colistin-Resistant *K. pneumoniae* Associated With UTI

Plazomicin, FDA approved new semisynthetic aminoglycoside for UTI, was reported to be efficacious against colistin-resistant *Enterobacteriaceae* with both plasmid-mediated *mcr1* and chromosomal mutation of *pmrAB* or *phoPQ* or *mgrB*, although 10% of tested *K. pneumoniae* isolates demonstrated resistance to this novel medication (16).

Meropenem-vaborbactam, an innovative carbapenem-β lactamase inhibitor and FDA-approved medication for severe UTIs, has been reported to be effective against drug-resistant *Enterobacteriaceae* from all over the world (18, 19). However, the efficacy of this new antibiotic was reported to be decreased among KPC-producing *K. pneumoniae* strains lacking outer membrane porins and overexpressing AcrAB efflux pump (48).

Cefiderocol is another promising novel siderophore cephalosporin against drug-resistant *K. pneumoniae* due to its enhanced outer membrane penetration ability using iron transport system and intrinsic effective antimicrobial activities (21). Nevertheless, resistance issues were recorded following 21 days of initiating cefiderocol therapy (49).

Although these newly discovered medications proved useful as rescue therapy at different stages of clinical trial, one of the most efficacious approaches for limiting the evolution of resistance to newer medications and tackling colistin-resistant *K. pneumoniae* is utilizing the combination therapy which combines two or more antimicrobial throughout a treatment course (50).

## Combination Antibiotic Therapies to Overcome Colistin-Resistant *K. pneumoniae* Associated With UTI

Colistin in combination with different classes of antibiotics displayed substantial synergistic effects in suppressing the growth of colistin-resistant *K. pneumoniae* isolates *in vitro* (23). Sulfadiazine in combination with colistin had a remarkable *in vitro* synergy against colistin-resistant *K. pneumoniae*, independent of underlying colistin resistance mechanisms (24). According to previous study, colistin permeabilization on Gram-negative outer membrane allowed minocycline, linezolid, fusidic acid, and levofloxacin to generate significant *in vitro* synergistic interactions in the treatment of colistin-resistant *Enterobacteriaceae* (25). Recent study reported that combination therapy comprising double carbapenems and colistin displayed significant synergistic bactericidal effects against colistin-resistant *K. pneumoniae in vitro* (26). Ceftazidime-avibactam, recently approved for treating UTI can also be explored as a potential combination treatment with avibactam to overcome colistin-resistance in *K. pneumoniae in vitro* (27). Synergistic combinations that have been discovered to be effective *in vitro* are needed to investigate further for *in vivo* efficacies, pharmacokinetic/ pharmacodynamic characteristics, and subsequent clinical trials to evaluate their therapeutic usefulness.

According to retrospective cohort evaluation, combined gentamicin and tigecycline was significantly correlated with higher treatment success rates and survival in patients with colistin-resistant *K. pneumoniae* (28). A multicenter prospective study revealed that addressing extensively and pandrug-resistant *K. pneumoniae* with colistin-fosfomycin combination was significantly related with favorable clinical outcomes in critically ill patients (29). In septic shock patients with colistin-resistant *K. pneumoniae*, recent prospective cohort study found that targeted combination therapy with gentamicin. fosfomycin and tigecycline was significantly related with lower mortalities (30). According to a prior case study, administration of double carbapenem treatment was proven to be beneficial in eliciting an effective therapeutic response with no recurrence in patients with pandrug-resistant *K. pneumoniae* bacteremia and UTI (31).

However, previous single-center retrospective study revealed that using different combination therapies for colistin- and carbapenem-resistant *K. pneumoniae* did not contribute to a substantial improvement in patients survival (51). Moreover, combination therapy involving multiple antibiotics has been attributed to the risks of drug resistance, toxicities, bacterial superinfections, higher costs, and probable antagonism (52). Reutilizing the currently prescribed non-antibiotic drugs in combination therapy is another plausible repurposing approach for managing colistin resistance in *K. pneumoniae* uropathogens (53).

## Non-antibiotic Drugs in Combination Therapies to Overcome Colistin-Resistant *K. Pneumoniae* Associated With UTI

FDA-approved Metal ions chelator - EDTA was demonstrated to perform as an efficacious adjuvant for colistin to reverse colistin resistance both in *in vitro* and *in vivo* catheter-related biofilm infections of colistin-resistant *K. pneumoniae* (36).

Azidothymidine is an approved antiretroviral drug, and it also exhibits antibacterial activities against *Enterobacteriaceae* by acting on bacterial DNA synthesis (32). Previous studies demonstrated significant synergistic activities of azidothymidine for retaining therapeutic efficacies of colistin in colistin-resistant *K. pneumoniae* that express *mcr-1*, both *in vitro* and *in vivo* (33, 34).

Antiprotozoal pentamidine has been demonstrated to have antibacterial activity against carbapenem and colistin-resistant *Enterobacteriaceae* isolates both individually and in combination with other antibiotics including aminoglycosides, tigecycline, doripenem, and rifampicin (37). Their effective perturbant actions on bacterial outer membrane help the combined antibiotics to produce significant synergistic activities for increasing bacterial clearance in internal organs and enhancing survival in a systemic mouse infection model of colistin resistant *Acinetobacter baumannii* with pEtn-mediated LPS modification similar to that conferred by *mcr-1* (38).

Mitotane is currently used chemotherapeutic agent for adrenocortical carcinoma. Whereas, mitotane monotherapy had limited antimicrobial activities, polymyxin B combined with mitotane therapy displayed substantial synergistic antibacterial effects for bacterial killing and impeding regrowth of polymyxin-resistant *K. pneumoniae* pathogens both *in vitro* and *in vivo* murine burn models due to permeabilization effects of polymyxin, which allows migration of mitotane into bacterial cells for its antibacterial effects (39).

Veterinary antihelminth closantel was observed to be ineffective when it was given as monotherapy in treating *A. baumanii* (40). However, with uncoupling oxidative phosphorylation activities of closantel, polymyxin was proved to recover its antimicrobial properties against polymyxin-resistant *A. baumannii* isolates and this combination drastically prevented the establishment of resistance in polymyxin-susceptible isolates (40).

Niclosamide, parasiticidal drugs for tapeworm infection, has previously been shown to increase negative surface charges of colistin-resistant *K. pneumoniae* with mutated *mgrB* and *pmrB* genes, allowing colistin to reactive against these clinical strains (41).

Additionally, colistin was reported to regain effectiveness against polymyxin-resistant *K. pneumoniae* when given in combination with membrane-active selective estrogen receptor modulators (SERM) such as tamoxifen, raloxifen, and toremifene by their combined synergistic effects in disrupting the outer membrane (42).

Antidepressants (amitriptyline, citalapram, and sertraline), antipsychotics (chlorpromazine and levopromazine), and statins (simvastatin) were discovered to possess synergistic activities with polymyxin in treatment of *K. pneumoniae*. Among these, citalopram, sertraline, and spironolactone were demonstrated to exert consistent synergistic effects in augmenting polymyxin activities (54).

## Natural Compounds in Combination Therapies to Overcome Colistin-Resistant *K. Pneumoniae* Associated With UTI

Natural compounds used for other treatment purposes have also demonstrated promising outcomes as a prospective combination therapy for overcoming colistin resistance. Potent *mcr1* inhibitory activities of natural compound - pterostilbene in combination with polymyxin as inhibitor-antibiotic combination significantly improved antimicrobial activities of polymyxin against different *mcr* variants-positive *Enterobacteriaceae*, resulting in lower bacterial-induced pathological damage in internal organs and better lifespan in treated mice (43).

Resveratrol, a natural chemical found in many plants, was discovered to augment colistin efficacy against a diverse panel of colistin-resistant *K. pneumoniae* strains with various resistance mechanisms involving the *mgrB* and *pmrB* mutations (44).

Previous research has also demonstrated combining eugenol and colistin showed a remarkable synergistic impact in lowering the colistin dose required to elicit antibacterial effects on colistin-resistant *E. coli* due to activities of eugenol in binding Mcr1 and suppressing *mcr1* expression (45, 55).

## Additional Therapies to Overcome Colistin-Resistant *K. Pneumoniae* Associated With UTI

Bacteriophage therapy is an additional therapeutic modality with favorable clinical outcomes that exploits the bacteriolytic activities of phage to target a variety of drug-resistant pathogens in infected individuals (10). In biofilm-producing colistin-resistant *K. pneumoniae* with *mcr1* and *mgrB* mutations, higher sensitivities to phage were also reported both *in vitro* and *in vivo*, owing to better adherence of negative-charge phage to altered LPS of this pathogen (46).

Nanocarrier strategies are revolutionary therapies for combating drug-resistant pathogens by increasing penetration and concentration of drug at the infection site through targeted delivery approaches (56). Nanoparticle encapsulation of colistin has also shown to have stronger colistin counteracting activities against planktonic and biofilms of drug-resistant *K. pneumoniae* as compared to providing free colistin (47).

Another feasible alternative is to modify urinary catheters with antifouling coatings including hydrogels, polytetrafluoroethylene, polyzwitterions, and polyethylene glycol which limit bacterial colonization and subsequent biofilm formation in catheter-associated UTI by their repulsive properties (57). Moreover, silver nanoparticles in hydrogel composite were efficient antimicrobial coating in providing significant inhibitory effects against *K. pneumoniae* uropathogens (58). Various research on biocidal catheters, such as gentamicin-coated, nitric oxide-coated, nitrofurazone-impregnated, antimicrobial peptide-coated, and phage-impregnated catheters, have shown promising results in minimizing the growth and biofilm development of UTI-causing *K. pneumoniae* pathogens (59).

## Conclusion

Increasing resistance to colistin in *K. pneumoniae* uropathogens due to chromosomal mutations and plasmid-mediated *mcr* genes result in chronic severe and recurrent UTI in clinical settings. Furthermore, they could serve as a source for pathogen dissemination in the healthcare environment, emphasizing the significance of discovering alternative therapeutic strategies to effectively combat colistin-resistant *K. pneumoniae* uropathogens. Although novel antibiotics including plazomicin, meropenem/vaborbactam, and cefiderocol have been demonstrated to be successful as rescue therapy for colistin-resistant *K. pneumoniae*, reports of emerging resistance to these newer antibiotics render them concerning for use as monotherapy. Instead of utilizing antibiotic monotherapy, combining two or more antibiotics or repurposing non-antibiotic medications or natural compounds in combination therapy is another promising approach for tackling colistin resistance in *K. pneumoniae*. Bacteriophage therapy, nanocarrier strategies and modification of urinary catheters are also designated to be used as future innovative treatment modalities for successful control of colistin-resistant *K. pneumoniae* uropathogens. While several *in vitro* and *in vivo* studies have revealed the potent therapeutic effects of various alternative strategies for addressing colistin-resistant *K. pneumoniae*, further clinical trial studies are required to investigate their therapeutic efficacies and safety in human patients.

## Author Contributions

AS: conception and writing the original draft of the manuscript. PH: conception, supervision, and writing the original draft of the manuscript. SA, SL-i, and NR: supervision, critical review, and editing of the manuscript. DW and TC: conception, supervision, critical review, and editing of the manuscript. All authors contributed to the article and approved the submitted version.

## Funding

AS was supported under the Chulalongkorn University Graduate Scholarship Program for ASEAN Countries. DW was supported by Chulalongkorn University (Second Century Fund- C2F Fellowship), and the University of Western Australia (Overseas Research Experience Fellowship). The sponsor(s) had no role in study design; in the collection, analysis, and interpretation of data; in the writing of the report; or in the decision to submit the article for publication.

## Conflict of Interest

The authors declare that the research was conducted in the absence of any commercial or financial relationships that could be construed as a potential conflict of interest.

## Publisher's Note

All claims expressed in this article are solely those of the authors and do not necessarily represent those of their affiliated organizations, or those of the publisher, the editors and the reviewers. Any product that may be evaluated in this article, or claim that may be made by its manufacturer, is not guaranteed or endorsed by the publisher.
